# Validation of the Childhood Trauma Questionnaire (CTQ) in the Context of Trauma-Focused Treatment

**DOI:** 10.1177/10775595251328611

**Published:** 2025-03-27

**Authors:** Chris M. Hoeboer, Nomi Bodor, Danielle A. C. Oprel, Rianne A. de Kleine, Maartje Schoorl, Agnes van Minnen, Willem van der Does

**Affiliations:** 1 Department of Clinical Psychology, 4496 Leiden University, Leiden, The Netherlands; 2 Department of Psychiatry, 1234 Amsterdam UMC Location University of Amsterdam, Amsterdam, The Netherlands; 3 PsyQ, 647179 Parnassia Groep, The Hague, The Netherlands; 4 169092 Youz, Parnassia Group, The Hague, The Netherlands; 5 Leids Universitair Behandel- en Expertise Centrum, 4496 Leiden University, Leiden, The Netherlands; 6 Behavioural Science Institute, Radboud University, Nijmegen, The Netherlands

**Keywords:** childhood trauma questionnaire, retrospective reports, trauma, child abuse, child neglect

## Abstract

**Background:** The Childhood Trauma Questionnaire (CTQ) is widely used, but retrospective self-report measures may be susceptible to bias especially in the context of pathology. Therefore, we aimed to validate the CTQ in the context of reduced psychopathology following trauma-focused treatment. **Methods:** We analyzed 149 outpatients with posttraumatic stress disorder (PTSD) related to childhood abuse. Participants received one of three variants of prolonged exposure. The CTQ was administered at baseline and six months later. The internal consistency of the CTQ was assessed using Cronbach’s alpha, inter-item and item-total correlations. Convergent validity was assessed with the clinician administered PTSD Scale for DSM-5 (CAPS-5). The consistency of CTQ scores over time was analyzed using linear mixed models and intra-class correlation coefficients. **Results:** Most CTQ subscales demonstrated high internal consistency and satisfactory inter-item and item-total correlations except for physical neglect and minimization/denial subscales. CTQ subscales physical and sexual abuse exhibited adequate convergent validity with the CAPS-5. None of the CTQ subscales mean score changed significantly from baseline to follow-up. Agreement between the baseline and follow-up assessment within-persons was moderate at item-level but good at subscale-level except for subscale minimization/denial. Minimization/denial at baseline and change in symptomatology during treatment were not significantly related to change in CTQ subscale scores. **Conclusions:** These findings support the use of the CTQ subscales to retrospectively assess childhood maltreatment.

## Introduction

Childhood maltreatment involves actions by a parent or caregiver that result in potential harm, or threat of harm, to the child (Gilbert et al., 2009). Childhood maltreatment encompasses physical abuse, emotional abuse, sexual abuse, physical neglect and emotional neglect. All these types of childhood maltreatment are common across the globe (Moody et al., 2018; Stoltenborgh et al., 2015). Individuals who have experienced childhood maltreatment may develop posttraumatic stress disorder (PTSD) in childhood or adulthood (Kessler et al., 2017, 2018). Adults with a history of childhood maltreatment are also at increased risk of developing various psychiatric disorders and symptoms such as depression, anxiety disorders, suicidality, dissociation, personality disorders, substance abuse and aggression (Briere et al., 2016; Carr et al., 2020; Gilbert et al., 2009; Johnson et al., 1999; Norman et al., 2012).

Since childhood maltreatment is more prevalent in treatment seeking individuals compared to the general population, yet may remain undisclosed without prompting, its routine assessment is of clinical relevance (Moody et al., 2018). In a systematic umbrella review it was concluded that while most types of childhood maltreatment are associated with adverse outcomes, specific forms of childhood maltreatment are strongly linked to distinct adverse long-term outcomes. For example, sexual childhood abuse is strongly related to the onset of depression, alcohol and drug use disorders and later perpetration of sexual abuse and risky sexual behavior. Emotional childhood abuse is strongly related to the onset of depression, bipolar disorder, psychosis and eating disorders. Physical childhood abuse is strongly related to the onset of alcohol and drug use disorders and perpetration of intimate partner violence in adulthood. Emotional childhood neglect is strongly related to alcohol and drug use disorders (Carr et al., 2020). Additionally, experiencing multiple forms of childhood maltreatment further elevates the risk of developing mental health problems (Carr et al., 2020; Morgan & Gayer-Anderson, 2016). While limited reviews have examined the relationship between childhood maltreatment and PTSD, individual studies have shown that emotional abuse is specifically associated with more severe PTSD symptoms (Egeland, 2009; Hoeboer et al., 2021; Nöthling et al., 2019; Rameckers et al., 2021). These findings underscore the importance of assessing type, severity and cumulative experiences of childhood maltreatment to better understand PTSD and comorbid psychopathology in adults.

Measuring childhood maltreatment often relies on retrospective self-report measures, which may be susceptible to bias. A systematic review identified eight studies investigating the stability of reported childhood maltreatment, all of which found discrepancies between assessments over time (Van Giezen et al., 2005). For example, a large birth cohort study from New Zealand revealed inconsistent reports of childhood sexual and physical abuse between two assessments of participants at ages 18 and 21 Fergusson et al. (2000). In about half of the instances, childhood trauma recalled during one assessment was not reported at another (Fergusson et al., 2000). Subsequent studies have confirmed these discrepancies in reported childhood maltreatment, even within a shorter time span. For example, about 30% of Dutch adults reported childhood sexual abuse on one occasion and not another, only 4–6 weeks apart (Langeland et al., 2015). The majority of these discrepancies involved adults who reported childhood sexual abuse on the first but not the second occasion. The main reasons participants provided for discrepancies in this study included a different interpretation of childhood maltreatment regarding the perpetrator (e.g., not a family member), age limit (e.g., after 18) and ambiguous experiences (harassment rather than abuse). Note that these studies did not use well validates questionnaires to assess the maltreatment and sometimes only one or a few items in a yes/no format. These findings underscore the importance of measuring childhood maltreatment with validated questionnaires including multiple items per abuse type. Nevertheless, even such questionnaires might show inconsistencies in reported maltreatment over time due to current symptomatology (Bernstein et al., 2003; Colman et al., 2016; Liebschutz et al., 2018; Maughan & Rutter, 1997; Reuben et al., 2016). For example, depression is associated with a more negative interpretation of childhood memories, which may affect recall of traumatic memories (Brewin et al., 1993; Colman et al., 2016; Gotlib & Joormann, 2010; Whalley et al., 2012). Research also suggests that PTSD can influence recall of traumatic events. For example, veterans with more severe PTSD symptoms after deployment tended to report more traumatic childhood memories compared to their reports before deployment (Engelhard et al., 2008; Southwick et al., 1997).

The Childhood Trauma Questionnaire (CTQ) is a widely used measure for retrospectively assessing childhood maltreatment. It distinctly assesses type and severity of childhood maltreatment, and its validity has been confirmed repeatedly (Bernstein et al., 2003; Thombs et al., 2009). The CTQ also included a minimization/denial domain which reflects potential denial of childhood maltreatment (example item ‘*When I was growing up I had the perfect childhood*’) and therefore might be indicative of underreporting or inconsistent reporting. Few studies have addressed the stability of the CTQ following psychotherapy. Given the concerns about self-reporting, and the potential for psychopathology to bias recall of childhood maltreatment, this is an important area of research. The only study addressing the effect of treatment on reports of childhood abuse with the CTQ, found that subscales for abuse remained stable pre- to post-treatment, but the neglect subscales did not (Paivio, 2001). Paivio (2001) found excellent test-retest correlation pre- to post-treatment for subscales physical, emotional and sexual abuse (*r* > .85) in survivors of childhood abuse. However, subscale physical neglect showed a good (*r* = .71) and emotional neglect a fair (*r* = .62) test-retest correlation. Paivio (2001) did not explore whether symptom improvements during treatment influenced reported physical or emotional neglect, so it is unclear what caused the instability of the neglect subscales. The author, however, posited that neglect subscales of the CTQ refer to less concrete events compared to abuse subscales and therefore might be more prone to changes in recall. Note that this study was not focused on patients with PTSD and did not examine trauma-focused treatment, so more research is needed to draw conclusions about the use of the CTQ in this population.

Specifically in patients with PTSD, concerns have been raised about potential underreporting of childhood maltreatment due to avoidance symptoms (e.g., Rameckers et al., 2021). Avoidance is one of the fifth edition of Diagnostic and Statistical Manual of Mental Disorders (DSM-5) PTSD criteria and encompasses internal avoidance (e.g., avoidance of thoughts related to the traumatic event) and external avoidance (e.g., avoidance of certain places or persons). These symptoms might hinder people from disclosing (the full extent of) childhood maltreatment. To date, however, no study assessed whether a reduction in PTSD symptoms during trauma-focused treatment is related to changes is recall.

The aim of the present study was to validate the Childhood Trauma Questionnaire in the context of reduced psychopathology following trauma-focused treatment. We assessed the internal consistency of the CTQ subscales and the convergent validity with reported index event of the Clinician-administered PTSD scale for DSM-5 (CAPS-5). Additionally, we assessed whether CTQ subscale scores were stable from baseline to a 6-month follow-up at group and individual levels and whether item scores were stable at an individual level. Finally, we investigated whether a change in psychopathology (PTSD, depression, dissociation and posttraumatic cognitions) from baseline to 6-month follow-up was related to a change in CTQ subscale scores and whether minimization/denial at baseline predicted change in CTQ subscale scores baseline to 6-month follow-up.

## Method

### Study Design and Procedures

**Figure 1.**
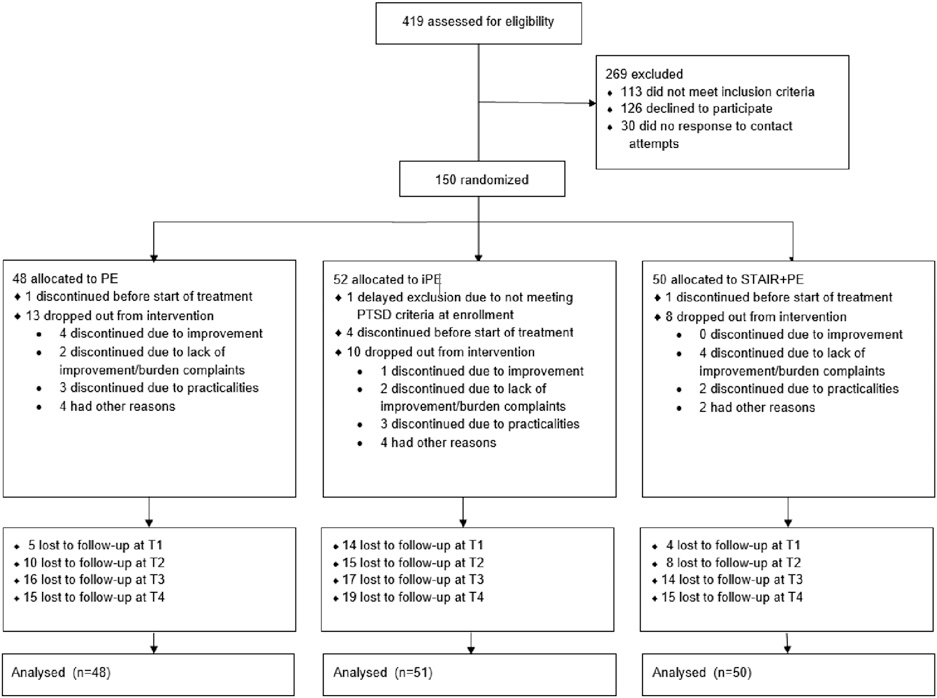
Flowchart of recruitment and follow-up process.

We used data from a trial investigating PTSD treatment for adults with childhood trauma: the IMPACT study. Full details of this study and primary outcomes are reported elsewhere (Oprel et al., 2021; Oprel et al., 2018). In brief, the study was a randomized controlled trial including three parallel conditions: Prolonged Exposure (PE), intensified Prolonged Exposure (iPE) and Skills Training in Affective and Interpersonal Regulation (STAIR) followed by PE (STAIR+PE). Participants were recruited from two outpatient clinics specializing in the treatment of PTSD located in The Hague and Rotterdam, the Netherlands. Inclusion criteria were: 1) age 18–65; 2) diagnosis of PTSD as established with the Clinician Administered PTSD Scale (CAPS-5, see measures section), and at least moderate severity of PTSD-symptoms (CAPS ≥ 26) with at least one specific memory for a traumatic event; 3) multiple traumata related to childhood sexual and/or physical abuse that occurred before 18 years of age, committed by a primary caretaker or an authority figure as index event; 4) sufficient fluency in Dutch to complete the treatment and research protocols. Exclusion criteria were: 1) involvement in a compensation case, legal procedures concerning admission or stay in the Netherlands 2) pregnancy; 3) severe non-suicidal self-injury (NSSI) which required hospitalization during the past three months; 4) severe suicidal behavior: a suicide attempt during the past three months or acute suicidal ideations with serious intent to die with a specific plan for suicide and preparatory acts; 5) severe disorder in the use of alcohol or drugs in last three months; 6) cognitive impairment (estimated IQ < 70); 7) changes in psychotropic medication in the two months prior to inclusion; and 8) engagement in any current psychological treatment. Participants provided written consent to participate (*N*=149). Figure 1 depicts the recruitment and follow-up process. The trial is registered at the clinical trials registry (clinicaltrials.gov), number NCT03194113 and was approved by the Medical Ethics Committee of Leiden University Medical Center (NL57984.058.16).

Before randomization, patients completed a baseline assessment and received a preparatory session with detailed information about the study procedures and the three treatments. Randomization was carried out by an independent researcher from Leiden University who used a computerized randomization sequence of permutated blocks of six patients, stratified by gender. Measurements were performed by trained and supervised interviewers, blind to treatment condition. Treatment was delivered by weekly supervised master's level therapists. Adherence to the treatment protocols was checked by independent observers, who rated randomly selected videotaped therapy sessions. For this study, we primarily used data collected at baseline and a 6-month follow-up which included the CTQ and measures of psychopathology. We also used data collected after 4, 8 and 16 weeks (post-treatment) of treatment which included all measures of psychopathology (but not the CTQ).

### Measures

#### Childhood Maltreatment

The short form of the Childhood Trauma Questionnaire (CTQ) was used to assess severity of self-reported childhood maltreatment (Bernstein et al., 1994; Thombs et al., 2009). The CTQ includes 28 items answered on five-point Likert scales (1 = never true, 2 = rarely true, 3 = sometimes true, 4 = often true, 5 = very often true). The CTQ consists of five subscales measuring five types of childhood maltreatment: emotional abuse (EA), emotional neglect (EN), physical abuse (PA), physical neglect (PN) and sexual abuse (SA). Each subscale is represented by five questions with a total subscale score ranging from 5 to 25 (higher scores indicate higher severity of childhood maltreatment). Total subscale score can be categorized into minimal, moderate, severe and extreme abuse. For descriptive purposes, we use cut-offs for severe to extreme abuse for EA (13 or higher), EN (15 or higher), PA (10 or higher), PN (10 or higher) and SA (8 or higher; Bernstein & Fink, 1998). In addition to the five subscales, the CTQ also contains a minimization/denial scale (three questions), that screens for the likelihood of underreporting traumatic experiences. Any question from this subscale scored as 5 (very often true) was coded as 1 and all other values as 0. Total score for the minimization/denial scale therefore ranges between 0–3. Previous studies showed excellent internal consistency of CTQ subscales PA (Cronbach’s = .91), EA (Cronbach’s alpha = .89), SA (Cronbach’s alpha = .95) and EN (Cronbach’s alpha = .91) but moderate internal consistency of subscale PN (Cronbach’s alpha = .63; Thombs et al., 2009). Further information on the reliability of the CTQ in the current sample is described in the results section.

#### PTSD Symptom Severity and A-Criterion

PTSD diagnosis and symptom severity were assessed with the Clinician-administered PTSD scale for DSM-5 (CAPS-5; Boeschoten et al., 2018; Weathers et al., 2018). The CAPS-5 includes 20 items answered on five-point Likert scales (0 = symptom is absent; 5 = symptom is extreme/invalidating). Total CAPS score is calculated by summing all items and ranges between 0–80 (higher scores indicate higher PTSD symptom severity). The CAPS-5 was administered in relation to the traumatic event (A-criterion) which caused most current burden to the participants. For the current study, it was coded whether the A-criterion included physical and/or sexual childhood abuse. Note that all participants reported childhood abuse as index event since this was an inclusion criterion for the original clinical trial (Oprel et al., 2021). Previous studies showed excellent internal consistency of the CAPS-5 (Cronbach’s alpha = .88) (Boeschoten et al., 2018; Weathers et al., 2018). In the present sample, the CAPS-5 had a moderate internal consistency (Cronbach’s alpha = .75) for the total severity score.

#### Depression Symptom Severity

The Beck Depression Inventory (BDI-II-NL) was used to measure the severity of depressive symptoms (Beck et al., 1996, 2002). The BDI-II-NL is a 21-item self-report depression inventory designed to assess symptom severity of depression. The questionnaire consists of 21 items about a particular symptom of depression with scores per item ranging from 0 to 3. Total scores can range from 0 and 63 with higher scores reflecting higher levels of depression. Reliability and validity of the BDI-II have been supported in previous studies (Beck et al., 2002; Osman et al., 2008). In the current sample, the BDI-II-NL had a high internal consistency (Cronbach’s alpha = .87).

#### Dissociative Symptoms

The Dissociative Experiences Scale (DES) was used to measure the degree to which participants experienced dissociative symptoms (Van IJzendoorn & Schuengel, 1996). The DES includes 28 items about the amount of time participants experienced symptoms each to be rated on a scale from 0%–100%. Total scores are calculated by averaging all items that range between 0–100 with higher scores reflecting more dissociative symptoms. Psychometric evaluation demonstrated a high internal consistency and good validity (Van IJzendoorn & Schuengel, 1996). In the present sample, the DES had a high internal consistency (Cronbach’s alpha = .95)

#### Posttraumatic Negative Cognitions

Posttraumatic negative cognitions were assessed using the Dutch version of the Posttraumatic Cognitions Inventory (PTCI; Foa et al., 1999; van Emmerik et al., 2006). The PTCI is a self-report measure and consists of 36 items of which 3 items are experimental: we therefore used 33 items, rated from 1 (‘totally disagree’) to 7 (‘totally agree’) with a total score of 33 items ranging from 33 to 231, with higher sores reflecting more posttraumatic negative cognitions. Psychometric evaluation demonstrated a high internal consistency and test-retest reliability (van Emmerik et al., 2006). In the current sample, the PTCI had a high internal consistency (Cronbach’s alpha = .94).

### Analyses

We agreed upon a statistical analysis plan before trial analysis (pre-registered at the Centre For Open Science; DOI 10.17605/OSF.IO/27TKB). We performed the analyses with SPSS statistical package version 27 (SPSS Inc, Chicago, IL) and R version 3.6.1 (R Core Team, 2018). Alpha was set at .05 for all analyses (two-tailed). We used package lme4 for modelling the linear mixed effects models (Bates et al., 2015). The models were estimated with random intercepts for persons and random slope effects of time to account for the dependency in the data within persons (Hox & Maas, 2002; Kato et al., 2005).

To answer the first question regarding the internal consistencies of the CTQ-subscales at baseline and the 6-month follow-up, we checked Cronbach’s alpha and item-total as well as inter-item correlations. We defined adequate internal consistency as: Cronbach’s alpha ≥.80; Cronbach’s alpha if item deleted increase ≤.05; item-total correlations ≥.30; inter-item correlation ≥.15 and ≤.50 (Clark & Watson, 2019).

We performed t-tests to check the convergent validity of the CTQ subscales sexual abuse and physical abuse with corresponding content of the DSM-5 A-criterion at baseline.

To investigate the consistency of the CTQ subscales baseline to 6-month follow-up at a group level, we performed five linear mixed effect models with CTQ subscales EA, EN, PA, PN and SA as the dependent variable and with assessment moment (time) as independent variable.

To check whether the consistency of the CTQ subscales (EA, EN, PA, PN and SA) and item scores (all 28 items) baseline to 6-month follow-up within persons was adequate, intra-class correlation estimates and their 95% confidence intervals were calculated based on a mean-rating (*k* = 2), absolute-agreement, 2-way mixed-effects model (Koo & Li, 2016).

We used a linear mixed effect model with CTQ subscale scores as dependent variable and with time, minimization/denial subscale at baseline and their interaction as independent variables to assess whether minimization/denial scores were related to a change in CTQ subscale scores. Moreover, we used linear mixed effect models with CTQ subscale scores as dependent variable and with A) time, change in PTSD symptoms and their interaction B) time, change in posttraumatic cognitions and their interaction C) time, change in depressive symptoms and their interaction and D) time, change in dissociative symptoms and their interaction as independent variables to assess whether there was a relationship between change in pathology and change in CTQ subscale scores. The assumptions of all analyses were met. Given the multiple outcome design (5 subscales of the CTQ) we applied Hommel multiple comparison correction to adjust *p*-values. We evaluated between-group effect sizes following the method of Feingold and *t*-to-*d* conversion using function lme-dscore from R package EMAtools (Bates et al., 2015).

## Results

**Figure 2.**
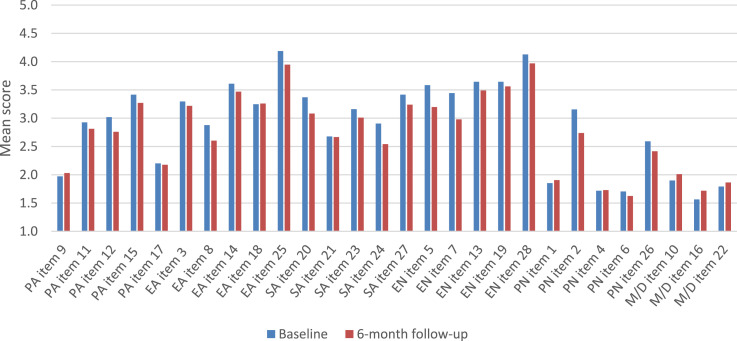
CTQ mean item scores at baseline (blue) and 6-month follow-up (red). CTQ subscales: PA = physical abuse; EA = emotional abuse; SA = sexual abuse; EN = emotional neglect; PN = physical neglect; M/D = Minimization/Denial

Participants’ demographic and baseline clinical characteristics are listed in Table 1. At baseline, 64% of the participants reported severe to extreme emotional abuse, 55% reported severe to extreme physical abuse, 65% reported severe to extreme sexual abuse, 59% reported severe to extreme emotional neglect and 34% reported severe to extreme physical neglect. All conditions led to a large decrease in PTSD symptoms from baseline to posttreatment (cohen’s *d* > 1.6) which was sustained during the follow-up without statistical differences between conditions. Figure 2 depicts the mean CTQ item scores at baseline and at the 6-month follow-up.

**Table 1.** Baseline Characteristics of the Participants.

Baseline characteristics, No (%)	*N* = 149
Gender (female)	114 (76.5)
Education (high)^a^	30 (20.1)
Cultural background (non-Western)^b^	65 (43.3)
DSM-5 A criterion CAPS trauma category (single or multiple)	
Childhood sexual abuse	108 (72.5)
Childhood physical abuse	93 (62.4)
Baseline characteristics, mean (SD)	
Age, mean (SD), years	36.9 (11.8)
Duration of PTSD, years	15.1 (12.5)
CAPS-5 score	41.4 (9.4)
BDI-II score	34.1 (10.4)
DES score	20.3 (16.1)
PTCI score	138.3 (36.7)

^a^high education = higher vocational education or university.

^b^non-Western cultural background = at least one parent was not born in a Western country.

### Internal Consistency

As listed in Table 2, the internal consistencies were high (Cronbach’s alpha >.85) at baseline and 6-month follow-up for all CTQ subscales except the physical neglect scale and the minimization/denial scale. Item-total correlations were all adequate except for item 4 (physical neglect) and item 22 (minimization/denial). Inter-item correlations were mostly adequate, but demonstrated many relatively high correlations between items (*r* > .50) in all subscales. Inter-item correlations for subscale physical neglect were relatively low between item 4 and 26 and item 4 and 1. See Table 3 for all inter-item and item-total correlations.

**Table 2.** Mean, Standard Deviation and Internal Consistency of CTQ Subscales at T0 (Baseline; *N = 149)* and at 6-Month Follow-up (*n* = 96).

CTQ	Baseline M(SD)	6-month follow-up M(SD)	Baseline Alpha	6-month follow-up Alpha
EA	17.2 (6.1)	16.5 (6.3)	.86	.88
EN	18.4 (5.3)	17.2 (5.6)	.86	.88
PA	13.5 (6.8)	13.1 (6.8)	.88	.90
PN	11.0 (4.3)	10.4 (4.1)	.65	.63
SA	15.5 (7.3)	14.5 (8.0)	.88	.92
M/D score above 1, No (%)	18 (12.1)	21 (21.9)	.47	.31

*Note.* CTQ = Childhood Trauma Questionnaire; EA = Emotional Abuse scale; EN = Emotional Neglect scale; PA = Physical Abuse scale; PN = Physical Neglect scale; SA = Sexual Abuse scale; M/D = Minimization/Denial scale; M = mean; SD = standard deviation; Alpha = Cronbach’s alpha.

**Table 3.** Internal Consistency of the CTQ Scales at Baseline: Item-Total and Inter-item Correlations.

	Item-total correlations	Inter-item correlations				
	CTQ PA	CTQ 9	CTQ 11	CTQ 12	CTQ 15	CTQ 17
CTQ 9	.65		.65	.49	.44	.62
CTQ 11	.82			.69	.73	.60
CTQ 12	.73				.67	.55
CTQ 15	.72					.53
CTQ 17	.68					
	CTQ SA	CTQ 20	CTQ 21	CTQ 23	CTQ 24	CTQ 27
CTQ 20	.88		.70	.87	.37	.87
CTQ 21	.70			.76	.24	.63
CTQ 23	.86				.36	.81
CTQ 24	.36					.39
CTQ 27	.81					
	CTQ PN	CTQ 1	CTQ 2	CTQ 4	CTQ 6	CTQ 26
CTQ 1	.45		.28	.10	.58	.24
CTQ 2	.48			.20	.34	.43
CTQ 4	.17				.20	.00
CTQ 6	.57					.34
CTQ 26	.37					
	CTQ EA	CTQ 3	CTQ 8	CTQ 14	CTQ 18	CTQ 25
CTQ 3	.71		.56	.73	.54	.50
CTQ 8	.67			.60	.57	.47
CTQ 14	.79				.63	.55
CTQ 18	.68					.49
CTQ 25	.59					
	CTQ EN	CTQ 5	CTQ 7	CTQ 13	CTQ 19	CTQ 28
CTQ 5	.63		.66	.45	.43	.56
CTQ 7	.73			.52	.56	.61
CTQ 13	.66				.62	.59
CTQ 19	.66					.58
CTQ 28	.72					
	CTQ M/D	CTQ 10	CTQ 16	CTQ 22		
CTQ 10	.36		.31	.26		
CTQ 16	.31			.18		
CTQ 22	.28					

### Convergent Validity

Patients who reported childhood physical abuse as A-criterion event during the CAPS interview, scored higher on the CTQ physical abuse subscale than patients who did not (*M =* 15.5*; SD =* 6.2 vs. *M =* 10.2*; SD =* 6.4*; p* < .001; *z* = 4.79). Patients who reported childhood sexual abuse during the CAPS interview scored higher on the CTQ sexual abuse subscale than those who did not (*M =* 18.3*; SD =* 6.1 vs. *M =* 8.3*; SD =* 4.7; *p* < .001; *z* = 7.17).

### Consistency over Time

We found that none of the CTQ subscale scores significantly changed from baseline to 6-month follow-up: physical abuse (*b* = .03; *t*(99) = .43; *p*_corrected_ = .67), sexual abuse (*b* = −.21; *t*(107) = = −1.64; *p*_corrected_ = .42), physical neglect (*b* = −.03; *t*(100) = −.53; *p*_corrected_ = .67), emotional abuse (*b* = −.08; *t*(101) = −1.03; *p*_corrected_ = .67) and emotional neglect (*b* = −.21; *t*(105) = −2.33; *p*_corrected_ = .11). On an individual level, the agreement within persons baseline to 6-month follow-up was moderate to good for all CTQ-scales (all scales ICC >.75) except for the M/D scale (ICC = .13). Agreement within persons from baseline to 6-month follow-up of the CTQ-items was moderate (CTQ PA item range .73–.84; CTQ SA item range .64–.77; CTQ PN item range .50–.78; CTQ EA item range .49–.77; CTQ EN item range .49–.72). See Table 4 for all intra-class correlation coefficients.

**Table 4.** Intra-class Correlation Coefficients of CTQ Subscales and Items From Baseline to 6-Month Follow-up.

	ICC	95% confidence interval
**CTQ PA**	.87	.81–.91
CTQ 9	.73	.62–.81
CTQ 11	.84	.78–.89
CTQ 12	.78	.69–.85
CTQ 15	.74	.59–.79
CTQ 17	.75	.65–.83
**CTQ SA**	**.77**	**.67–.84**
CTQ 20	.73	.62–.81
CTQ 21	.70	.59–.79
CTQ 23	.76	.66–.83
CTQ 24	.64	.51–.75
CTQ 27	.76	.66–.83
**CTQ PN**	**.88**	**.83–.92**
CTQ 1	.60	.45–.71
CTQ 2	.50	.33–.64
CTQ 4	.67	.54–.77
CTQ 6	.78	.69–.85
CTQ 26	.59	.44–.71
**CTQ EA**	**.83**	**.76–.89**
CTQ 3	.77	.67–.84
CTQ 8	.70	.59–.79
CTQ 14	.76	.66–.86
CTQ 18	.70	.48–.79
CTQ 25	.49	.32–.63
**CTQ EN**	**.75**	**.65–.83**
CTQ 5	.72	.59–.81
CTQ 7	.49	.32–.63
CTQ 13	.58	.43–.70
CTQ 19	.51	.35–.65
CTQ 28	.65	.51–.75
**CTQ M/D**	**.13**	**−.07–.32**
CTQ 10	−.10	−.30–.10
CTQ 16	−.04	−.24–.16
CTQ 22	.64	.51–.75

We found no significant association between minimization/denial at baseline and change in any CTQ subscale: physical abuse (*b* = .15; *t*(97) = .95; *p*_corrected_ = .98), sexual abuse (*b* = .47; *t*(102) = 1.89; *p*_corrected_ = .24), physical neglect (*b* = −.003; *t*(97) = −.03; *p*_corrected_ = .98), emotional abuse (*b* = .30; *t*(98) = 1.98; *p*_corrected_ = .20) and emotional neglect (*b* = −.02; *t*(100) = −.12; *p*_corrected_ = .98). Moreover, changes in PTSD severity from baseline to 6-month follow-up were not significantly related to the changes in CTQ subscales: physical abuse (*b* = −.03; *t*(111) = −.59; *p*_corrected_ = .93), sexual abuse (*b* = −.01; *t*(133) = −.09; *p*_corrected_ = .93), physical neglect (*b* = .01; *t*(117) = .35; *p*_corrected_ = .93), emotional abuse (*b* = .06; *t*(116) = 1.07; *p*_corrected_ = .93) and emotional neglect (*b* = .07; *t*(131) = 1.11; *p*_corrected_ = .93). We also found no significant relationship between change in BDI, DES or PTCI scores from baseline to 6-month-follow-up and change in any CTQ subscale.

## Discussion

We examined the validity and reliability of the Childhood Trauma Questionnaire (CTQ) in the context of changes in psychopathology during trauma-focused treatment in a large and diverse clinical population with a history of childhood trauma. The results largely confirm the stability of CTQ scores before and after trauma-focused therapy.

At baseline, the CTQ demonstrated excellent internal consistency across most subscales, with the exception of the physical neglect and minimization/denial subscales. For physical neglect, specifically item 1 ‘*When I was growing up I didn’t have enough to eat’* and item 4 ‘*When I was growing up my parents were too drunk or high to take care of the family’* showed low correlations with other items in the subscale. This finding aligns with previous research (Bernstein et al., 1994; Gerdner & Allgulander, 2009; Paivio & Cramer, 2004; Peng et al., 2023; Thombs et al., 2009). It has been suggested that item 4 of the CTQ in particular may need to be revised or removed (Peng et al., 2023). Since the issues with this subscale are reported across different cultures, this appears to be a general problem and we recommend future studies to investigate this further. For subscale minimization/denial, the internal consistency was mediocre. While previous validation studies of the CTQ often ignored this subscale (Bernstein et al., 2003; Thombs et al., 2009), some studies reported better internal consistency of the minimization/denial subscale with a Cronbach’s alpha around .70 (Gerdner & Allgulander, 2009; MacDonald et al., 2015). Notably, in the current study minimization/denial was rarely endorsed (12.1% at baseline) compared to 28% in previous clinical samples and 42% in community samples (MacDonald et al., 2016). This might be related to the current patient population which all sought treatment for PTSD related to childhood abuse. Those minimizing/denying childhood abuse might rarely seek out treatment focused on childhood abuse and therefore this subscale might not be relevant and reliable in the current population.

The convergent validities of the CTQ subscales physical and sexual abuse in relation to their CAPS-5 A-criteria were supported. This supports the use of a self-report measure to assess childhood maltreatment. Furthermore, we found no significant changes in CTQ subscale scores from baseline to 6-month follow-up. This finding is in line with previous research in a sample of adult survivors of childhood abuse (Paivio, 2001). Likewise, recall of childhood traumatic events remained stable pre- to post-treatment in a study of fifty patients with borderline personality disorder (Kremers et al., 2007).

On an individual level, we found a moderate to good agreement between the CTQ scores at baseline and 6-month follow-up, except for the Minimization/Denial subscale which proved generally unstable. On an item level, items from the (physical, sexual and emotional) abuse subscales remained stable (only exception being item 25 ‘*When I was growing up I believe that I was emotionally abused.’*) while severity scores of a few items of the (physical and emotional) neglect subscales demonstrated a low agreement between the baseline and follow-up measurement, especially, item 2 ′*When I was growing up I knew that there was someone to take care of me and protect me’,* item 7 ‘*When I was growing up I felt loved*’, item 13 ‘*When I was growing up people in my family looked out for each other*.’, item 19 ‘*When I was growing up people in my family felt close to each other*.’, and item 26 *‘When I was growing up there was someone to take me to the doctor if I needed it.*’ (all ICCs below .6). This corresponds with previous research into the CTQ which also found that neglect subscales were less stable over time compared to abuse subscales (e.g., Paivio, 2001). This may be explained by the different nature of abuse and neglect: child neglect refers to the absence of certain events, making it harder to identify as incidents than for example sexual or physical abuse (Hildyard & Wolfe, 2002). Neglect is also a heterogeneous construct concerning rather dissimilar negative experiences referring to the omission of caretaking behavior, whereas abuse tends to be a harmful act committed against a child (Mulder et al., 2018). This chronic and passive/omissive nature of childhood neglect may make it hard to categorize the severity of the neglect in an answer option. Alternatively, the framing of the questions of the neglect subscale might have introduced bias or errors. To likely prevent bias in response tendencies, the questionnaire included both negatively and positively framed questions. However, only seven items in the CTQ are framed positively and most of these items demonstrated a low agreement between baseline and follow-up and were part of the neglect subscales. This imbalance in question framing could have introduced bias or errors. Additionally, the positively framed questions were distributed randomly throughout the questionnaire rather than grouped together, which may have led to accidental misinterpretations. Future research should explore whether the CTQ yields better results when all items are framed negatively or when the question framing is more balanced. However, the low agreement on these items did not lead to systematic changes in the total subscale scores, since these scores remained consistent over time. This indicates that it is important to rely in CTQ subscale scores rather than item scores when using the questionnaire. More generally, it is important to measure experiences of childhood (physical/emotional) neglect with multiple items to avoid inconsistencies over time.

We found no relationship between endorsement of minimization/denial at baseline and change in CTQ outcome. Given the low internal consistency and consistency over time of this subscale, it is unsurprising that it did not have added value in the current study. Note that other studies investigating the minimization/denial subscale of the CTQ also found that it did not serve as a response bias index (MacDonald et al., 2015). Additionally, we found no relationship between change in pathology and change in CTQ scores. This finding is in line with previous studies that showed retrospective self-reports of childhood maltreatment to remain stable in the context of reduced psychopathology (Goltermann et al., 2023; Kremers et al., 2007; Paivio, 2001). A recent study investigated the relationship between changes in depressive symptoms and changes in CTQ scores in a mixed clinical sample (primarily depressed patients). An increase in CTQ scores pre- to post-treatment was related to a reduction in depressive symptoms (Ernst et al., 2023). This does not seem to be explained by sample characteristics, since many patients in the current study also met criteria for a depressive disorder. Nor can it be explained by treatment outcome since treatment of PTSD also improved depression scores in the current study (Hoeboer et al., 2021). However, participants in the current study were recruited in a specialized psychotrauma center and therefore were probably aware of the connection between their traumatic past and current mental health symptoms from the start of treatment while participants in the study by Ernst et al. (2023) were recruited in a clinic for psychosomatic medicine and psychotherapy and might have become more aware of the relevance of their traumatic past throughout the course of treatment. Hence, more research on the influence of (mood) state and retrospective recall of childhood maltreatment is important especially in the context of depression.

Our findings imply that clinicians can effectively use the CTQ to assess type and severity of childhood maltreatment in patients with PTSD since symptoms of PTSD, depression, dissociation and posttraumatic cognitions do not affect CTQ scores. This implies that the experience of maltreatment, in terms of both type and severity, is unlikely to change over the course of treatment. Furthermore, it is unlikely that childhood maltreatment is repressed to the extent that someone does not report it on the CTQ. Similarly, it is unlikely that childhood maltreatment is overreported due to current symptomatology. Previous studies have reported inconsistencies in reports of childhood maltreatment when measured using single dichotomous items. This implies that the CTQ might be more reliable owing to its format (several items per type of maltreatment and an interval scale). Hence, we recommend that clinicians use the CTQ rather than rely on a limited number of self-made questions about childhood maltreatment. Moreover, our findings indicate that research into types of childhood maltreatment in relation to treatment outcomes is relevant. However, we recommend to use subscale scores rather than single items. In situations where single items are used (e.g. when designing a short-form screener), we recommend to carefully select the most reliable items since we found considerable differences in reliability between items.

There are a few limitations to this study. Firstly, the current sample size did not allow for factor analyses to confirm the factor structure of the CTQ. Secondly, although the CTQ remained stable, we cannot confirm whether the consistently recalled information itself is actually correct. Lastly, participants of the current study were part of a trial focused on the effectiveness of PE for patients with at least moderately severe PTSD related to childhood maltreatment. This resulted in a study sample with many participants reporting severe childhood maltreatment. We recommend future studies to investigate whether current results generalize to other samples.

Despite these limitations, our study is the first to investigate the stability of the CTQ in a large and diverse sample of people suffering from childhood abuse-related PTSD. It is also the first study that investigated the stability of the CTQ at an individual level as well as at group level in patients who received trauma-focused treatment. The present study confirms the stability of the CTQ in the context of reduced psychopathology after trauma-focused treatment in a sample of treatment-seeking people with childhood abuse-related PTSD. Based on some inconsistencies on the individual item-level, we recommend to rely on subscale scores and encourage future studies to investigate whether the CTQ performs better when questions are all framed similarly (with a higher score reflecting more severe maltreatment). Furthermore, we suggest that the CTQ items may be grouped by dimension; after all, in a clinical interview one also does not skip from one type of abuse to the next and back.

Despite these inconsistencies, the CTQ subscale scores remained stable. Therefore, the current study supports the usability of the CTQ for clinical and empirical purposes as a reliable measure of self-reported childhood maltreatment.
